# Evanescent Wave-Assisted Symmetry Breaking of Gold Dipolar Nanoantennas

**DOI:** 10.1038/srep32194

**Published:** 2016-09-01

**Authors:** Jhen-Hong Yang, Kuo-Ping Chen

**Affiliations:** 1 Institute of Photonic System, National Chiao Tung University, 301 Gaofa 3rd Road, Tainan 71150, Taiwan; 2 Institute of Imaging and Biomedical Photonics, National Chiao Tung University, 301 Gaofa 3rd Road, Tainan 71150, Taiwan

## Abstract

Symmetry-breaking and scattering cancellation were observed in the dark-mode resonance of dipolar gold nanoantennas (NAs) on glass substrates coupled with oblique incidence and total internal reflection. With the assistance of evanescent waves, the coupling efficiency was twice as strong when the incidence angle was larger than the critical angle. The Hamiltonian equation and absorption spectra were used to analyze the hybridization model of symmetric dipolar gold NAs. The antibonding mode could be coupled successfully by both transverse-magnetic (TM) and transverse-electric (TE) polarizations to NAs when the dimers orientation is parallel to the propagation direction of evanescent waves.

Localized surface plasmon resonance (LSPR) for metallic nanoparticle dimers has attracted considerable attention in recent years^1^. The plasmonic resonance in the metal-dielectric interface has been investigated for many applications, such as bio-detection^2^, fluorescence enhancement^3^, active optoelectronic components^4^, metamaterials^5^, and surface-enhanced Raman scattering^6^. Nanoantennas (NAs), i.e., metallic nanoparticle dimers, have exhibited different resonance modes, including the dipolar bonding mode and antibonding mode^7^. Numerous researchers have attempted to analyze the plasmonic coupling mode with the particle-dimer-like array structure because of the strong field enhancements^8^,^9^.

The interactions between NAs and incident electromagnetic waves have been investigated. Plasmonic coupling is interpreted according to the plasmon hybridization model introduced by Nordlander *et al*.^10^,^11^,^12^,^13^,^14^. For identical nanoparticle dimers, the resonance wavelength varies among different coupling modes owing to the corresponding phase of the electric displacement field caused by transverse-magnetic (TM) and transverse-electric (TE) polarization fields. For metallic nanoparticle dimers, the in-phase (out-of-phase) response of two dipoles is called the bonding (antibonding) mode or bright (dark) mode, as in molecular orbital theory^12^,^13^. The bonding mode can be coupled easily by normal incidence, but in symmetric structures of NAs, the antibonding mode would be hardly coupled by normal incidence. There have been researches discussed that the antibonding mode can be coupled by a metal-insulator-metal (MIM) structure^15^, an asymmetric structure^16^, or with different metals^17^; however, fabrication for those structures is difficult, and the coupling efficiency is lower than that of the general symmetric boning mode. Despite the difficulty of coupling energy into the antibonding mode of identical NAs, the antibonding-mode resonance of NAs attracts considerable attention owing to the slower radiative decay and narrower linewidths. The antibonding-mode resonance of NAs can be predicted according to the plasmonic Hamiltonian theory. The eigenvalue of plasmonic quadratic Hamiltonian equation for representing two identical coupled localized surface plasmons (LSPs) is^17^


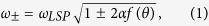


where


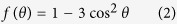


Here, *α* is the interaction coefficient, which is mainly depended on the distance of two nanoparticles. The LSP frequency of single nanoparticle is *ω*_*LSP*_, and *θ* is the angle formed by the polarization of the nanoparticles and the dimer axis joining the two nanoparticles.

For *θ* = 0, the low-energy (high-energy) mode with frequency *ω*_−_(*ω*_+_) can be considered as the in-phase (out-of-phase) motion of the two nanoparticles. For *θ* = *π*/2, the − and + modes correspond to the out-of-phase and in-phase motions, respectively.

Studies have been conducted to predict the coupling modes and explore the characteristics of different modes by theoretical analysis. However, theoretical results cannot provide information about the coupling efficiency in hybridization models. To further investigate the coupling efficiency, finite-element method (FEM) analysis and experimental results are necessary. In this work, the oblique incidence is chosen to couple energy into symmetric gold NAs on glass substrates, and the effects of different incidence angles are compared.

By solving the plasmonic quadratic Hamiltonian equation, four kinds of coupling modes are indicated in Fig. 1. The different directions of the dipole resonance are also shown. The resonance wavelengths of different plasmonic coupling modes, such as the transverse bonding mode or the longitudinal antibonding mode, can be calculated by theoretical analysis. The four types of plasmonic coupling modes shown in Fig. 1 are the longitudinal-bonding (LB) mode, the longitudinal-antibonding (LA) mode, the transverse-bonding (TB) mode, and the transverse-antibonding (TA) mode, which correspond to the wavelengths of 780, 677, 698, and 749 nm, respectively. The interaction coefficient (*α*) of nanoantennas is 0.037 in the model.

In Fig. 1, the resonance wavelength of the LA mode (red arrow near 677 nm) is shorter than that of the LB mode (black arrow near 780 nm) because the potential energy of the LA mode is high. In contrast, the resonance wavelength of the TA mode is longer than that of the TB mode because the potential energy of the TA mode is low. The results clearly show that the electric potential changes according to the dipole direction. In addition, the label at 728.5 nm indicates the resonance wavelength of single particle case. Equation 1 indicates that the single-particle resonance frequency (*ω*_*LSP*_) should always be in the middle of the LA (TA) and LB (TB) modes, which means that there is no hybridization.

To ensure the accuracy of the theoretical analysis, the NAs arrays are fabricated and the absorption spectra in FEM simulation are compared at wavelengths ranging from 600 to 880 nm. Electron-beam lithography is applied to fabricate arrays of gold NAs on a 15-nm indium-tin oxide (ITO)-coated glass substrate. Polymethylmethacrylate is used as the photoresist in the lithography process. As shown in the scanning electron microscopy (SEM) images of Fig. 2, highly symmetric particles are fabricated. The square particle dimers have x and y dimensions of 98 and 100 nm, respectively, with a periodicity of 400 nm in both the x and y directions. The thickness of the NAs is 36.3 nm, and the gap between the two paired particles in a unit cell is ~32 nm. In the measurement, the reflectance spectra from 600 to 900 nm of the NA array are recorded. The absorption spectra in Fig. 2 are calculated as (1-R-T), where R stands for the reflectance and T stands for the transmittance. In the simulation, the loss factor of gold in the Drude model is 3. The refractive index of the substrate is 1.52 (glass), the surrounding medium is water (n = 1.33), and the ITO layer is not considered. Comparing Fig. 1 with Fig. 2g–i reveals that the theoretical analysis fits well with the FEM modeling, not only for the case of a single particle but also for NAs. In Fig. 2g, because the symmetry is not broken by the oblique incidence in the TM transverse setting, there is only one resonance wavelength, ~700 nm, which corresponds to the TB mode in Fig. 1. In Fig. 2h, the blue shift of the resonance wavelength is observed when the incidence angle is larger than 30°. At a normal or small-angle oblique incidence (<30°), the resonance wavelength is 780 nm, which corresponds to the LB mode in Fig. 1. When the incidence angle is larger than 30°, another resonance mode arises, which occurs at 680 nm and corresponds to the LA mode in Fig. 1. In Fig. 2i, the resonance wavelength is 728.5 nm, which corresponds to the case of a single particle in Fig. 1. According to the analysis, good agreement is observed not only for the resonance wavelength but also for the plasmonic coupling mode.

Interestingly, in Fig. 2h, when incidence angle is larger than the critical angle, a strong enhancement of the coupling efficiency in the antibonding mode is observed. The reason for this enhancement is explained by Fig. 3. Because the NAs are thin, emphasis should be placed on the x component of the electric fields near the interface. In Fig. 3, different incident angles and corresponding near-field distributions of the electric displacement field are shown. For the left case in Fig. 3, under normal incidence, the electric field is parallel to the interface between glass and water, which means that the NAs can receive 100% of the intensity from the incident electromagnetic wave to couple with the plasmonic LB mode, as shown in the corresponding near-field distribution. For the middle case in Fig. 3, when the incidence angle is increased, the x component of the electric field decreases. However, when the incidence angle is larger than the critical angle, the transmitted light changes into an evanescent wave. For the evanescent wave, the wavefront is longitudinal, and the electric displacement field at the interface becomes stronger than that under normal incidence^18^,^19^. In Fig. 3, comparing the electric displacement field at the interface for incidence angles of 40° and 72° reveals that the intensity of the electric dipole moment at 72° is twice as strong as that of the LA mode at 40°. The absorption (1-R-T) spectra in two-dimensional (2D) mapping (Fig. 2h) and the near-field distribution indicate that the antibonding mode in NAs can be strengthened by evanescent waves.

The top and bottom spectra in Fig. 4 show the simulation and experimental results, respectively. The reason for showing only the reflectance spectra is that the transmittance is negligible when the incidence angle is larger than the critical angle. As shown in the figure, the trends in the simulation and experimental spectra agree well. The reflectance dip in the simulation is narrower than that in the experiment. This difference between the simulation and experiment may be due to the fabrication imperfections of the sample.

Figure 5a,b show the near-field distribution of the LB and LA modes, respectively. For the antibonding mode, the LSPR strength is proportional to the absorptivity because the net dipole moment tends to be zero. In contrast, the bonding mode is a bright mode, which means that the LSPR strength is proportional to the scattering. In Fig. 2h, the corresponding resonance mode is the antibonding mode; thus, the absorptivity is enhanced when the incidence angle is larger than the critical angle.

In Fig. 5, the scattering cross section, absorption cross section, and total extinction cross section spectra for the bonding mode and antibonding mode of a single nanoantenna are analyzed by Mie theory like FEM modeling. Here, the total extinction cross section is the sum of the scattering and absorption cross section. The scattering cross section includes the light scattered in all directions and the absorption cross section relates to the energy absorbed by the nanoantenna. In Fig. 5c, the net dipole of the bonding mode is nonzero; thus, the scattering cross section dominates the total extinction cross section. Conversely, when the net dipole of the antibonding mode is zero, the absorption cross section dominates the total extinction cross section, at the resonance wavelength. In the antibonding mode of longitudinal resonance, the scattering cross section is suppressed by a factor of five compared with that in the bonding mode.

In Fig. 6, the TE polarization for the resonance of the transverse NAs (Fig. 6a) is analyzed. In Fig. 1, the resonance wavelength of the TB mode is 698 nm, which corresponds to Fig. 6b, and that of the TA mode—749 nm—corresponds to Fig. 6c. The enhancement of the electric displacement field of the NAs by the evanescent wave is observed by comparing the extinction cross section and the near-field distribution in Fig. 6.

In previous research, the antibonding mode was usually coupled by TM longitudinal-type waves because of the property of breaking symmetry^20^,^21^,^22^. However, in our study, the antibonding mode could be coupled by oblique TE polarization transverse-type waves, demonstrating that TE transverse waves can also break the symmetry of NAs when the dimers orientation is transverse to the polarization of incident field.

For a clear comparison of the different plasmonic coupling modes, the information of Figs 5 and 6 are arranged in Table 1. Here, the full widths at half maximum (FWHMs) of the antibonding modes are smaller than those of the bonding mode. In addition, the ratio of the absorption cross section to the total extinction cross section of the antibonding modes are larger than those of the bonding mode. The reason is because antibonding modes are with slower radiative decay than bonding modes^10^. These results, which also correspond to the bright (bonding) mode and dark (antibonding) mode in Fig. 1, agree well with the physical meaning of the “bright” and “dark” modes and the differences in the radiative decay and linewidths.

The hybridization model of plasmonic NAs and the coupling resonance wavelengths can be predicted by solving the plasmonic Hamiltonian equation. Different types of coupling modes can be found by changing the incidence angles. In addition, by examining the reflectance spectra and near-field distribution, the enhancement of the coupling efficiency for the antibonding mode of NAs under the evanescent wave can be explained without the complex molecular-orbital theory. In addition to a simulation and theoretical analysis, an experiment was performed to improve the credibility of this study. The bonding (bright) mode and antibonding (dark) mode were coupled in both TM-longitudinal type and TE-transverse type polarization wave in an NA array. In the future, the antibonding mode can be applied to improve the sensitivity of sensors or in quantum optics because of its high quality factor and slower radiative decay.

## Additional Information

**How to cite this article**: Yang, J.-H. and Chen, K.-P. Evanescent Wave-Assisted Symmetry Breaking of Gold Dipolar Nanoantennas. *Sci. Rep.* **6**, 32194; doi: 10.1038/srep32194 (2016).

## Figures and Tables

**Figure 1:**
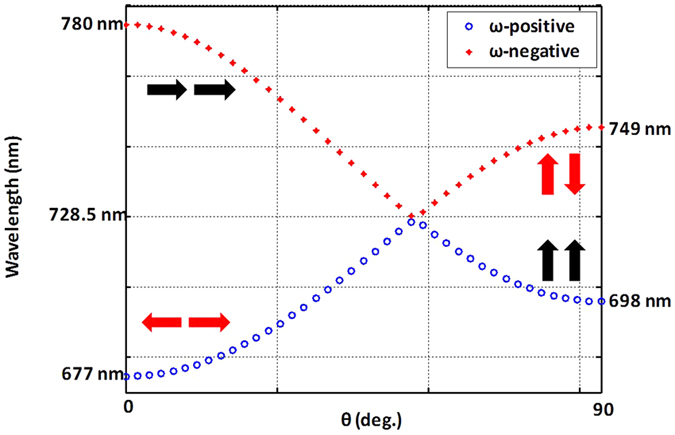
Theoretical analysis results obtained by solving the eigenvalue of plasmonic quadratic Hamiltonian equation. Resonance wavelengths are calculated by Eq. (1). According to Eq. (1), two frequencies can be observed. The higher frequency is called ω-positive (circle), and the other one is called ω-negative (star).

**Figure 2:**
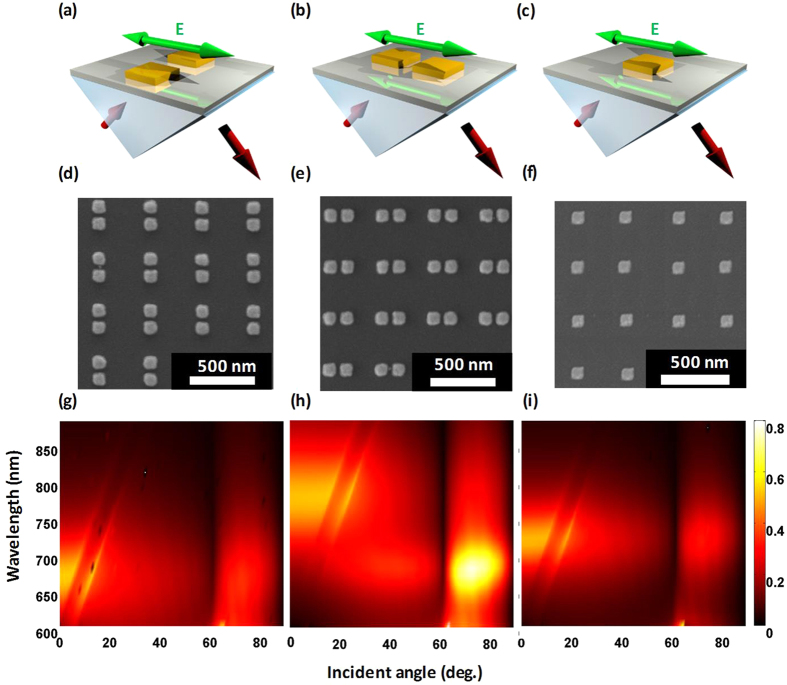
(**a**–**c**) Oblique incidence framework; (**d**–**f**) SEM images; and (**g**–**i**) 2D mapping absorption (1-R-T) spectra from FEM simulation with TM-polarized incidence light for incidence angles ranging from 0° to 89° and wavelengths ranging from 600 to 880 nm of (**a**,**d**,**g**) TM transverse-type antennas; (**b**,**e**,**h**) TM longitudinal-type antennas; and (**c**,**f**,**i**) a TM-type single particle. The red arrows indicate the direction of the light.

**Figure 3:**
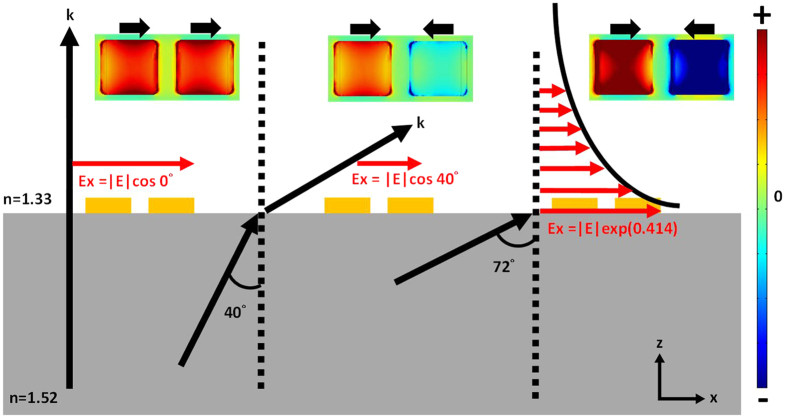
Normal incidence, oblique incidence, and total internal reflection of NAs. The x-component of the electric field at the interface (z = 0) is indicated as Ex for different incidence angles. The distribution of the electric displacement field of the NAs at 670 nm is shown at the top for each case.

**Figure 4:**
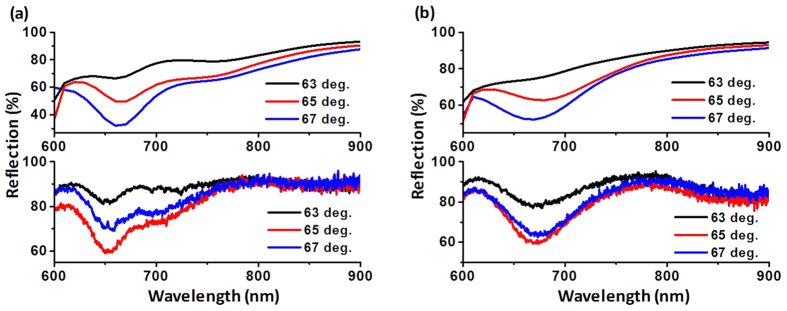
TM-polarization reflectance spectra of NAs with different incidence angles, which are all larger than the critical angle: (**a**) longitudinal-type and (**b**) transverse-type. The top and bottom spectra show the simulation and experimental results, respectively.

**Figure 5:**
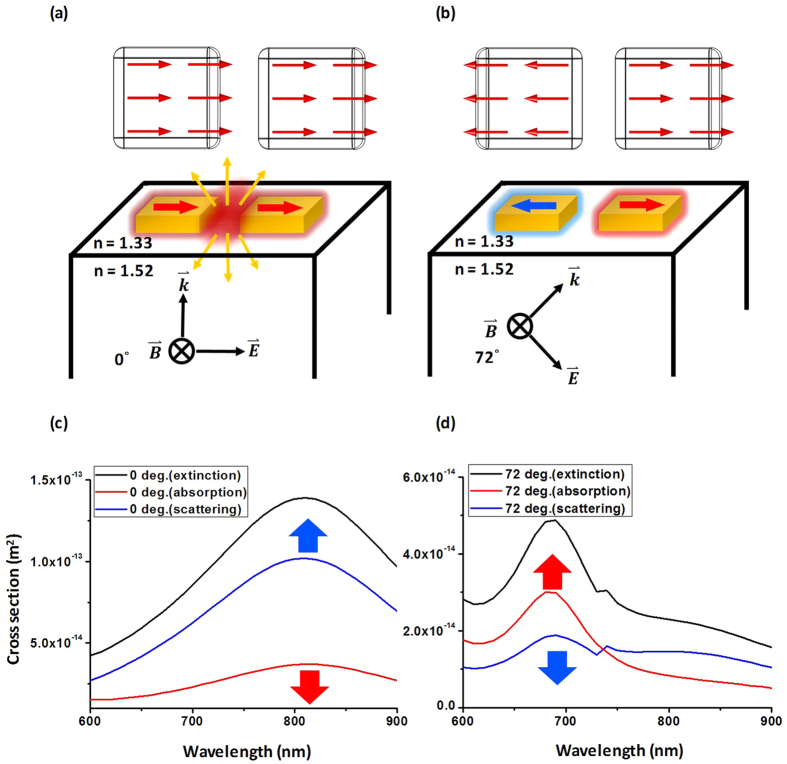
Simulation results for near-field distribution and dipole distribution sketch map of (**a**) bonding mode (bright mode) and (**b**) antibonding mode (dark mode). The extinction cross-section spectra of the scattering part and absorption part for (**c**) 0° and (**d**) 72° from the FEM simulation.

**Figure 6:**
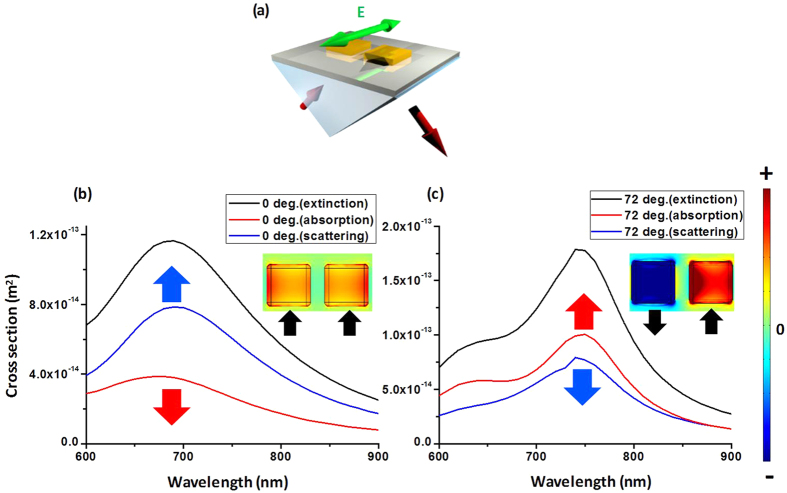
(**a**) Framework of oblique TE polarization for transverse NAs. FEM simulation results for the extinction cross-section spectra and near-field distribution of the electric displacement field for (**b**) the TB mode and (**c**) the TA mode at incidence angles of 0° and 72°, respectively.

**Table 1:** Resonance wavelengths, ratio of scattering cross section and absorption cross section to total extinction cross section and FWHM in different resonance modes of NAs.

	Longitudinal		Transverse	
	0°-bonding	72°-anti.	0°-bonding	72°-anti.
λ_res_ (nm)	780	677	698	749
σ_sca/_σ_ext_	0.73	0.38	0.67	0.46
σ_abs/_σ_ext_	0.27	0.62	0.33	0.54
FWHM (nm)	198.8	59.98	176.83	106.6

## References

[b1] Chen Y.-H., Chen K.-P., Shih M.-H. & Chang C.-Y. Observation of the high-sensitivity plasmonic dipolar antibonding mode of gold nanoantennas in evanescent waves. Appl. Phys. Lett. 105, 031117 (2014).

[b2] Naik G. V. . Titanium nitride as a plasmonic material for visible and near-infrared wavelengths. Optical Materials Express 2, 478–489 (2012).

[b3] Kinkhabwala A. . Large single-molecule fluorescence enhancements produced by a bowtie nanoantenna. Nature Photonics 3, 654–657 (2009).

[b4] Knight M. W., Sobhani H., Nordlander P. & Halas N. J. Photodetection with active optical antennas. Science 332, 702–704 (2011). 21551059 10.1126/science.1203056

[b5] Schurig D. . Metamaterial electromagnetic cloak at microwave frequencies. Science 314, 977–980 (2006). 17053110 10.1126/science.1133628

[b6] Hatab N. A. . Free-standing optical gold bowtie nanoantenna with variable gap size for enhanced Raman spectroscopy. Nano Lett. 10, 4952–4955 (2010). 21090585 10.1021/nl102963g

[b7] Yang S.-C. . Plasmon hybridization in individual gold nanocrystal dimers: direct observation of bright and dark modes. Nano letters 10, 632–637 (2010). 20058898 10.1021/nl903693v

[b8] Rechberger W. . Optical properties of two interacting gold nanoparticles. Optics Communications 220, 137–141, 10.1016/s0030-4018(03)01357-9 (2003).

[b9] Chen W.-L. . The Modulation Effect of Transverse, Antibonding, and Higher-Order Longitudinal Modes on the Two-Photon Photoluminescence of Gold Plasmonic Nanoantennas. ACS nano 8, 9053–9062 (2014). 25207747 10.1021/nn502389s

[b10] Pelton M. & Bryant G. W. Introduction to metal-nanoparticle plasmonics. Vol. 5 (John Wiley & Sons, 2013).

[b11] Nordlander P., Oubre C., Prodan E., Li K. & Stockman M. Plasmon hybridization in nanoparticle dimers. Nano Lett. 4, 899–903 (2004).

[b12] Wang H., Brandl D. W., Nordlander P. & Halas N. J. Plasmonic nanostructures: artificial molecules. Accounts of chemical research 40, 53–62 (2007). 17226945 10.1021/ar0401045

[b13] Prodan E., Radloff C., Halas N. J. & Nordlander P. A hybridization model for the plasmon response of complex nanostructures. Science 302, 419–422 (2003). 14564001 10.1126/science.1089171

[b14] Prodan E. & Nordlander P. Plasmon hybridization in spherical nanoparticles. The Journal of chemical physics 120, 5444–5454 (2004). 15267418 10.1063/1.1647518

[b15] Dmitriev A., Pakizeh T., Käll M. & Sutherland D. S. Gold–silica–gold nanosandwiches: tunable bimodal plasmonic resonators. Small 3, 294–299 (2007). 17199248 10.1002/smll.200600409

[b16] Shegai T. . A bimetallic nanoantenna for directional colour routing. Nat. Commun. 2, 481 (2011). 21934665 10.1038/ncomms1490 PMC3195252

[b17] Brandstetter-Kunc A., Weick G., Weinmann D. & Jalabert R. A. Decay of dark and bright plasmonic modes in a metallic nanoparticle dimer. Phys. Rev. B 91, 035431 (2015).

[b18] Bliokh K. Y., Bekshaev A. Y. & Nori F. Extraordinary momentum and spin in evanescent waves. Nat. Commun. 5 (2014). 10.1038/ncomms4300 24598730

[b19] Bekshaev A. Y., Bliokh K. Y. & Nori F. Mie scattering and optical forces from evanescent fields: A complex-angle approach. Opt. Express 21, 7082–7095 (2013). 23546090 10.1364/OE.21.007082

[b20] Panaro S. . Dark to bright mode conversion on dipolar nanoantennas: a symmetry-breaking approach. ACS Photonics 1, 310–314 (2014).

[b21] Osberg K. D. . Systematic Study of Antibonding Modes in Gold Nanorod Dimers and Trimers. Nano Lett. 14, 6949–6954 (2014). 25411044 10.1021/nl503207j

[b22] Chu M.-W. . Probing bright and dark surface-plasmon modes in individual and coupled noble metal nanoparticles using an electron beam. Nano Lett. 9, 399–404 (2008). 19063614 10.1021/nl803270x

